# Supplementary material for: Cost-Effectiveness of Strategies Addressing Environmental Noise: A Systematic Literature Review
Source: Int J Environ Res Public Health. 2025 May 21;22(5):803. doi: 10.3390/ijerph22050803 (PMC12110787; doi:10.3390/ijerph22050803)
Supplement: Supplementary file 1 [file ijerph-22-00803-s001.zip › ijerph-3575628-supplementary.pdf]

Supplementary Material S1

Medline (Pubmed)

Concept	Query
noise	"Noise" [MeSH] OR "noise" [tiab] OR "Sound" [MeSH] OR "sound" [tiab] OR "noise pollut*" [tiab] OR "sound pollut*" [tiab] OR "noise lev*" [tiab] OR "sound lev*" [tiab] OR "ambient noise pollut*" [tiab] OR "ambient sound pollut*" [tiab]
full economic evaluation / health effect	"Cost-Benefit Analysis" [Mesh] OR "cost-benefit" [tiab] OR "cost utilit*" [tiab] OR "costutilit*" [tiab] OR "cost and benefit*" [tiab] OR "costs and benefit*" [tiab] OR "benefit and cost*" [tiab] OR "benefits and cost*" [tiab] OR "marginal analys*" [tiab] OR "economic evaluation*" [tiab] OR "Cost-Effectiveness Analysis" [Mesh] OR "cost-effectiveness" [tiab] OR "ICER" [tiab] OR "incremental cost-effectiveness ratio" [tiab] OR "incremental cost-utility ratio" [tiab] OR "ICUR" [tiab] OR "cost minimization" [tiab] OR "costminimization" [tiab] OR "cost efficien*" [tiab] OR "costefficien*" [tiab] OR "health economic" [tiab] OR "healtheconomic" [tiab] OR "Quality-Adjusted Life Years"[Mesh] OR "Quality adjusted life year*" [tiab] OR "QALY*" [tiab] OR "healthy years equivalent*" [tiab] OR "Disability-Adjusted Life Years" [Mesh] OR OR "disability-adjusted life year*" [tiab] OR "DALY* " [tiab] OR "Life Expectancy" [Mesh] OR "life expectanc*" [tiab] OR "years of potential life lost" [tiab] OR "health impact" [tiab] OR "health effect*" [tiab] OR "health gain" [tiab] OR "health outcome*" [tiab] OR "health benefit*" [tiab] OR "health implication*"[tiab] OR "Comparative Effectiveness Research" [MeSH] OR "comparative effectiveness research" [tiab] OR "effectiveness" [tiab]
strategy	"intervention*" [tiab] OR "strateg*" [tiab] OR "reduction" [tiab] OR "mitigation*" [tiab] OR "assessment*" [tiab] OR "policy"[tiab] OR "policies"<br>[tiab] OR "abatement*"[tiab] OR "management"[tiab]

Web of Science

Concept	Query
noise	TS=(“noise” OR “sound” OR “noise pollut*” OR "sound pollut*" OR “noise lev*” OR “sound lev*” OR "outdoor noise pollut*" OR “ambient sound pollut*”)
full economic evaluation / health effect	TS=("cost-benefit*" OR "cost utilit*" OR “costutilit*” OR "cost and benefit*" OR "costs and benefit*" OR "benefit and cost*" OR "benefits and cost*" OR "marginal analys*" OR "economic evaluation*" OR "cost-effectiveness analysis" OR "cost-effectiveness" OR “ICER” OR "incremental cost-effectiveness ratio" OR "cost minimization" OR “costminimization” OR "cost efficien*" OR “costefficien*” OR "health economic" OR “healtheconomic” OR "quality-adjusted life year*" OR QALY* OR "healthy years equivalent*" OR "disability-adjusted life year*" OR “DALY*” OR "life expactanc*" OR "years of potential life lost" OR "health impact" OR "health effect*" OR "health gain" OR "health outcome*" OR "health benefit*" OR "health implication*" OR “comparative effectiveness research” OR “effectiveness”)
strategy	TS=(“intervention*” OR “strateg*” OR “reduction” OR “mitigation*” OR “assessment*” OR “policy“ OR “policies” OR “abatement*” OR “management”)

Embase

Concept	Query
noise	‘Noise’/exp OR ‘Sound’/exp OR ‘noise’:ti,ab,kw OR ‘sound’:ti,ab,kw OR 'noise pollut*':ti,ab,kw OR ‘sound pollut*’:ti,ab;kw OR 'noise lev*':ti,ab,kw OR 'sound lev*':ti,ab,kw OR 'ambient noise pollut*':ti,ab,kw OR 'ambient sound pollut*’pollut*':ti,ab,kw
full economic evaluation / health effect	'Cost-Benefit Analysis'/exp OR 'cost-benefit':ti,ab,kw OR 'cost utilit*':ti,ab,kw OR costutilit*:ti,ab,kw OR 'cost and benefit*':ti,ab,kw OR 'costs and benefit*':ti,ab,kw OR 'benefit and cost*':ti,ab,kw OR 'benefits and cost*':ti,ab,kw OR 'marginal analys*':ti,ab,kw OR 'economic evaluation*':ti,ab,kw OR 'Cost-Effectiveness Analysis'/exp OR 'Cost-Effectiveness':ti,ab,kw OR 'cost Effectiveness':ti,ab,kw OR 'incremental cost-effectiveness ratio':ti,ab,kw OR ‘ICER’:ti,ab,kw OR ‘incremental cost-utility ratio’:ti,ab,kw OR ‘ICUR’:ti,ab,kw OR 'cost minimization':ti,ab,kw OR ‘costminimization’:ti,ab,kw OR 'cost efficien*':ti,ab,kw OR ‘costefficien*’:ti,ab,kw OR 'health economic':ti,ab,kw OR healtheconomic:ti,ab,kw OR 'Quality-Adjusted Life Years'/exp OR 'quality adjusted life year*':ti,ab,kw OR QALY*:ti,ab,kw OR 'healthy years equivalent*':ti,ab,kw OR 'Disability-Adjusted Life Years'/exp OR 'disability-adjusted life year*':ti,ab,kw OR DALY*:ti,ab,kw OR 'Life Expectancy'/exp OR 'life expactanc*':ti,ab,kw OR 'years of potential life lost':ti,ab,kw OR 'health impact':ti,ab,kw OR 'health effect*':ti,ab,kw OR 'health gain':ti,ab,kw OR 'health outcome*':ti,ab,kw OR 'health benefit*':ti,ab,kw OR 'health implication*':ti,ab,kw OR ‘Comparative Effectiveness Research’/exp OR ‘comparative effectiveness research’:ti,ab,kw OR ‘effectiveness’:ti,ab,kw
strategy	Intervention*:ti,ab,kw OR strateg*:ti,ab,kw OR reduction:ti,ab,kw OR mitigation*:ti,ab,kw OR assessment*:ti,ab,kw OR policy:ti,ab,kw OR policies:ti,ab,kw OR abatement*:ti,ab,kw OR management:ti,ab,kw

Supplementary Material S2

first author	publication year	journal	volume	pages	reason for exclusion
Piao	2022	Science of the Total Environment	842	156846	outcome
Schiavoni	2022	Noise Mapping	9	89-108	study design
Yang	2021	Systems and Information Engineering Design Symposium		1-6	intervention
Rossi	2020	Environment international	145	106126	outcome
Rohacs	2020	Transport	35	193-202	study design
Vogiatzis	2019	Noise Mapping	7	87-98	study design
McNally	2018	Integrated environmental assessment and management	14	22-31	intervention
Ross	2018	Transportation Research Record	2672	144-153	study design
Ortega	2017	Proceedings of the Institution of Mechanical Engineers, Part F	232	1800-1813	study design
Perez	2017	Journal of Transport & Health	4	316-324	intervention
Siciliano	2016	European Transport Research Review	8	23	intervention
Hammer	2014	Environmental health perspectives	122	115-119	study design
Moliner	2014	Dyna	89	77-84	study design
Wolfe	2014	Transport Policy	34	102-108	study design
Venegas-Sanchez	2013	Gaceta sanitaria	27	233-240	language

Hammer, M.S.; Swinburn, T.K.; Neitzel, R.L.: Environmental noise pollution in the United States: developing an effective public health response. *Environ Health Perspect* **2014**, *122*, 115-119. 10.1289/ehp.1307272

McNally, A.D.; Fitzpatrick, A.G.; Mirchandani, S.; Salmon, M.; Edwards, D.A.: CERCLA-linked environmental impact and benefit analysis: Evaluating remedial alternatives for the Portland Harbor Superfund Site, Portland, Oregon, USA. *Integr Environ Assess Manag* **2018**, *14,* 22-31. 10.1002/ieam.2000

Moliner, E.; Vidal, R.; Franco, V.: Garraín, D.: A method to assess the impact of road transport noise within the framework of life cycle assessment. *Dyna* **2014**,: *89*,8. https://doi.org/10.6036/5804

Ortega, A.; Blainey, S.; Preston, J.: Installation of under sleeper pads on ballasted railway tracks: An economic analysis of their potential implementation. *Proceedings of the Institution of Mechanical Engineers Part F-Journal of Rail and Rapid Transit* **2017**, *232,*14. https://doi.org/10.1177/0954409717748811

Pérez, K.; Olabarria, M.; Rojas-Rueda, D.; Santamariña-Rubio, E.; Borrell, C.; Nieuwenhuijsen, M.: The health and economic benefits of active transport policies in Barcelona. *J Transp Health* **2017**, *4*, 9. https://doi.org/10.1016/j.jth.2017.01.001

Piao, Z.; Heutschi, K.; Pieren, R.; Mikhailenko, P.; Poulikakos, L.D.; Hellweg, S.: Environmental trade-offs for using low-noise pavements: Life cycle assessment with noise considerations. *Sci Total Environ* **2022**, *842*, 156846. 10.1016/j.scitotenv.2022.156846

Rohacs; J.; Rohacs, D.: Total impact evaluation of transportation systems. *Transport* **2020**, *35*, 10. https://doi.org/10.3846/transport.2020.12640

Ross, J.C.; Arnoldy, M.; Evans, J.: New Hampshire Department of Transportation Statewide Noise Barrier Study. *Transportation Research Record* **2018**, *2672*, 10. https://doi.org/10.1177/0361198118797188

Rossi, I.A.; Vienneau, D.; Ragettli, M.S.; Flückiger, B.; Röösli, M.: Estimating the health benefits associated with a speed limit reduction to thirty kilometres per hour: A health impact assessment of noise and road traffic crashes for the Swiss city of Lausanne. *Environ Int* **2020**, *145,* 106126. 10.1016/j.envint.2020.106126

Schiavoni, S.; D'Alessandro, F.; Baldinelli, G.; Turrioni, C.; Schenone, C.; Borelli, D.; Marsico, G.: Guidelines for a common port noise impact assessment: the ANCHOR LIFE project. *Noise Mapping* **2022**, *9*, 20. https://doi.org/10.1515/noise-2022-0006

Siciliano, G.; Barontini, F.; Islam, D.M.Z.; Zunder, T.H.; Mahler, S.; Grossoni, I.: Adapted cost-benefit analysis methodology for innovative railway services. *European Transport Research Review* **2016**, *8*. https://doi.org/10.1007/s12544-016-0209-5

Venegas-Sánchez, J.; Rivadeneyra-Sicilia, A.; Bolívar-Muñoz, J.; López-Fernández, L.A.; Martín-Olmedo, P.; Fernández-Ajuria, A.; Daponte-Codina, A.; Ruiz-Fernández, J.; Artundo-Purroy, C.: [Health impact assessment of the San Fernando street renewal project in Alcalá de Guadaíra (Seville, Spain)]. *Gac Sanit* **2013**, *27*, 233-240. 10.1016/j.gaceta.2012.08.002

Vogiatzis, K.; Dimitriou, D.; Gerolymatou, G.; Konstantinidis, A.: Strategic noise mapping in Athens International Airport: A tool for balanced approach & health effects evaluation. *Noise Mapping* **2019**, *7*, 12. 10.1515/noise-2020-0008

Wolfe, P.J.; Yim, S.H.L.; Lee, G.; Ashok, A.; Barrett, S.R.H.; Waitz, I.A.: Near-airport distribution of the environmental costs of aviation. *Transport Policy* **2014**, *34,* 7. https://doi.org/10.1016/j.tranpol.2014.02.023

Yang, C.; Marshall, A.A.; Mott, J.H.: A Novel Platform Design for Aircraft Noise Impact Assessment. *Systems and Information Engineering Design Symposium (SIEDS)* **2021**, 6. 10.1109/SIEDS52267.2021.9483734
